# Diagnostic Value of CD25, CD69, and CD134 on Tuberculosis-Specific Antigen-Stimulated CD4+ T Cells for Tuberculous Pleurisy

**DOI:** 10.1155/2023/5309816

**Published:** 2023-09-27

**Authors:** Hanlu Shi, Liping Yang, Fujie Zhang, Yu Zhou, Yonglie Zhou

**Affiliations:** ^1^Laboratory Medicine Center, Department of Clinical Laboratory, Zhejiang Provincial People's Hospital (Affiliated People's Hospital, Hangzhou Medical College), Hangzhou 310014, China; ^2^School of Medical Technology and Information Engineering, Zhejiang Chinese Medical University, Hangzhou 310053, China; ^3^The Quzhou Hospital of Wenzhou Medical University, Quzhou People's Hospital, Quzhou 324000, China; ^4^Qian Xi Nan Hospital of Traditional Chinese Medicine, Qian Xi Nan Buyei and Miao Autonomous Prefecture, Guizhou 562499, China; ^5^Key Laboratory of Biomarkers and in vitro Diagnosis Translation of Zhejiang Province, Hangzhou, Zhejiang 310063, China

## Abstract

Rapid and accurate methods for the diagnosis of tuberculous pleurisy (TP) are urgently needed. Activation markers of tuberculosis (TB)–reactive T cells are considered promising for the diagnosis of active TB (ATB). Different activation indexes may play different roles in the progression of TB, but there are few reports on T cell activation indicators, except for HLA-DR. Hence, we evaluated the expression of early (CD25 and CD69) and late (CD134) activation markers on TB antigen-stimulated CD4+ T cells in populations with different TB infection status and investigated their diagnostic value for ATB, particularly, for TP. Moreover, we compared the differences in the diagnostic efficacy among the indexes from peripheral blood (PB) and pleural fluid (PF) for TP. The expression of each activation marker was significantly increased in TB-infected populations (patients with ATB and latent TB infection vs. healthy individuals; patients with TP vs. non-TP) and was significantly higher in the PF than in the PB of patients with TP. The diagnostic performance of the coexpressed activation markers was superior to that of single expression markers in the differential diagnosis of ATB and non-TB, with CD25+CD134+ showing the best diagnostic efficiency (AUC: 0.93, 95% CI, 0.87–0.99; sensitivity: 86.7%, 95% CI, 72.5%–94.5%; and specificity: 94.0%, 95% CI, 82.5%–98.4%). Except for TB-IGRA, the activation indexes were more accurate than conventional laboratory methods for ATB diagnosis. In addition, the expression of CD25+CD134+ in PB and PF was the best values for differential diagnosis of TP and NTP, with AUCs of 0.87 (95% CI, 0.77–0.96) and 0.95 (95% CI, 0.90–1.00), respectively. Our study provides information on the diagnostic value of different activation markers for TB and shows that the expression of CD25+CD134+ on CD4+ T cells in PF can serve as a potential marker for TP diagnosis.

## 1. Introduction

Tuberculosis (TB) is the infectious disease with the highest mortality rate, nearly 10 million people worldwide had TB in 2021 [1]. Meanwhile, nearly 2 billion people have latent tuberculosis infection (LTBI), which manifests as a sustained immune response to *Mycobacterium tuberculosis* (Mtb) antigen stimulation but without clinical evidence of active tuberculosis (ATB) [2]. On average, 5%–10% of LTBI cases develop ATB [3]. ATB can be further divided into pulmonary TB (PTB) and extrapulmonary TB (EPTB) according to the site of infection. Tuberculous pleurisy (TP) is the second most common form of EPTB after lymph node TB [4, 5]. However, the diagnosis of TP is often challenging because of the paucity of Mtb in pleural fluid (PF) and sometimes requires invasive procedures to obtain pleural tissue for histological, microbiological, or analytical examinations [6]. Therefore, rapid and accurate methods to facilitate the diagnosis of TP are urgently required [7].

CD4+ T cells play a critical role in the immune response against Mtb. The primary role of CD4+ T cells is to coordinate and regulate the immune response upon antigen recognition through releasing cytokines [8–11], but the widely used interferon-gamma (IFN-*γ*) release assay (IGRA) results in false negatives due to impaired immunity [12] and cannot distinguish between ATB and LTBI [13]. In contrast, the activation [14, 15], differentiation [16], and cytokine coexpression profiles [17] of TB-reactive T cells have shown promising diagnostic applications in ATB diagnosis. Among them, the activation phenotype expressed in Mtb-specific T cells appears to be particularly outstanding [18]. For example, CD27 has been associated with active disease and tissue destruction in TB [19]; CD137 has been used to identify reactive T cells in response to TB antigens [20]; CD38, an early immune marker of T cell activation, was found to be significantly superior to CD27 in the accurate diagnosis of TB [21]; HLA-DR, usually highly expressed during the late stage of T cell activation [22, 23], is believed to have the potential to distinguish patients with ATB from LTBI individuals [24]. Nevertheless, T cells activation could also be indicated by other activation markers, such as CD25, CD69, and CD134. An interesting blood-based study reported that when T cells were stimulated by antigens, CD25 and CD134 expressions were upregulated [25]. The combined upregulation of CD25 and CD134 allows the identification of antigen-specific CD4+ T cells in TB and various other infections [26]. CD25, the alpha chain of IL-2 receptor, is transiently highly expressed in T cells after activation by T cells receptor. As an early marker of lymphocyte activation, CD25 plays a critical importance in the proliferation, survival, and function of T cells [27, 28]. CD134, a member of the tumor necrosis factor receptor superfamily, mainly plays a role in the later stages of T cell proliferation and survival maintenance [29]. Sauzullo et al. [30] found that, after stimulation with Mtb-specific antigens, the expression of CD134 in the peripheral blood (PB) of patients with ATB was higher than that of LTBI individuals and healthy controls. In addition, CD69, a type II glycoprotein with a C-type lectin-like domain, is an early surface marker upregulated after T cell activation [31]. A study showed that the expression of CD69+CD4+ T cells in patients with ATB and tuberculin skin test-positive healthy individuals was significantly higher than that in tuberculin skin test-negative healthy controls [32].

Different activation biomarkers may play different roles in ATB progression. However, the value of various activation indexes in the diagnosis of different TB infection status has not been fully elucidated. Though TP is considered a suitable model for assessing local protective cellular immune responses against Mtb infection [33], the activation-associated phenotype of T cells induced by Mtb-specific antigens in PF is still unclear.

Inspired by the above evidence, we proposed the use of different activation markers to improve the diagnostic performance of ATB, with an emphasis on TP identification. The expression of the activation markers CD25, CD69, and CD134 on TB-specific CD4+ T cells was evaluated using a simple technique to stimulate PB/PF with TB-specific antigens. Given that the combination of diagnostic biomarkers is useful in specific situations, the value of activation marker coexpression in the diagnosis of ATB, especially TP, was also investigated.

## 2. Materials and Methods

### 2.1. Study Populations and Ethics Statements

**Figure 1:**
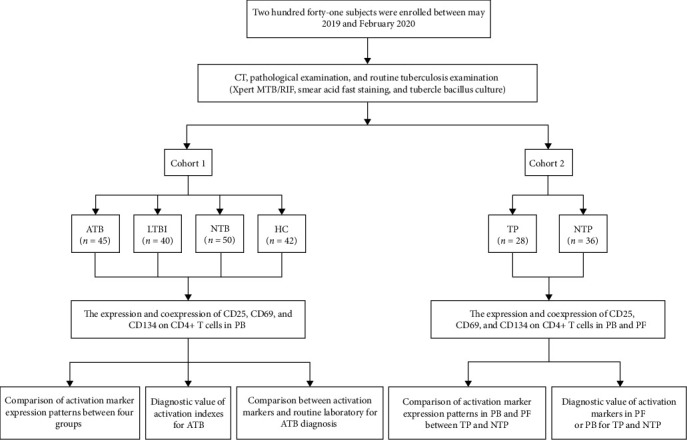
Experimental flowchart of this study. ATB = active tuberculosis; LTBI = latent tuberculosis infection; NTB = nontuberculosis; HC = healthy control; TP = tuberculous pleurisy; NTP = nontuberculous pleurisy; PB = peripheral blood; PF = pleural fluid.

The current study was conducted between May 2019 and February 2020. A total of 151 participants were recruited from Zhejiang Quzhou People's Hospital, whereas 90 were enrolled from Zhejiang Provincial People's Hospital. The enrollees were divided into two cohorts according to whether PB was the only sample studied or PF and PB were simultaneously analyzed. Cohort 1 was used to compare the diagnostic value of activation markers in PB for TB, and participants were categorized as follows: (1) ATB: patients with ATB defined according to the presence of TB-related clinical symptoms and the evidence of ATB in etiologic, pathologic, and imaging analyses; (2) LTBI: those individuals who were positive for TB-IGRA but had no clinical evidence of ATB [34]; (3) non-TB disease (NTB): this category included patients with different kinds of tumors (such as acute myeloid leukemia and gastric malignancy), infectious diseases (such as chronic hepatitis B, pneumonia, and infectious peritonitis), autoimmune diseases (such as rheumatoid arthritis, systemic lupus erythematosus, and subacute thyroiditis), and chronic diseases (such as hypertension, diabetes, and coronary heart disease); and (4) healthy controls (HC): healthy people with TB-IGRA negative results that were also excluded from having underlying diseases by CT and other clinical examinations. Cohort 2 was used to further evaluate the value of activation markers in PB and PF in the diagnosis of TP, which consisted of the following: (1) TP: patients diagnosed with TP after clinical manifestations, CT, and bacteriological examination and (2) non-TP (NTP): patients excluding TP but with inflammatory or malignant PF. The flowchart in Figure 1 synthesized the experimental strategy followed in this study. This study was approved by the ethics committee of Zhejiang Provincial People's Hospital, Affiliated People's Hospital, Hangzhou Medical College, Hangzhou, Zhejiang, China (no. 2019KY224). Informed consent was obtained from all participants.

### 2.2. Specimen Collection and Pretreatment

Fresh PB samples were collected from all participants in sodium heparin tubes, whereas fresh 10–20 mL PF samples were collected in parallel from those participants belonging to cohort 2. The white blood cell count in the PF was adjusted to 2–10 × 10^6^ cells/mL before analysis. Subsequently, PB and PF samples were split into three tubes (1 mL each tube): a negative control tube containing RPMI-1640 medium only, a test tube containing 5 *μ*g/mL ESAT-6 and CFP-10 (Wantai, Beijing, China), and a positive control tube containing 10 *μ*g/mL phytohemagglutinin (PHA). All cells were simultaneously costimulated with 1 *μ*g/mL anti-CD28 and anti-CD49d antibodies (BD Biosciences). The tubes were then incubated at 37 °C in 5% CO_2_ for 24 hr.

### 2.3. Flow Cytometry Procedures

After incubation, 100 *μ*L PB or PF was stained with 2 *μ*L anti-CD4 FITC, anti-CD25 PerCP/Cy5.5, anti-CD69 PE/Cy7, and anti-CD134 PE (BioLegend company) and kept away from light at room temperature for 15 min. Then, 450 *μ*L NH_4_Cl hemolysin was added to each tube and kept away from light at room temperature for 10 min. After centrifugation, the cells were fixed in 450 *μ*L PBS buffer and analyzed by flow cytometry (FC500, Beckman Coulter company). At least 50,000 events were acquired in lymphocyte gates.

**Figure 2:**
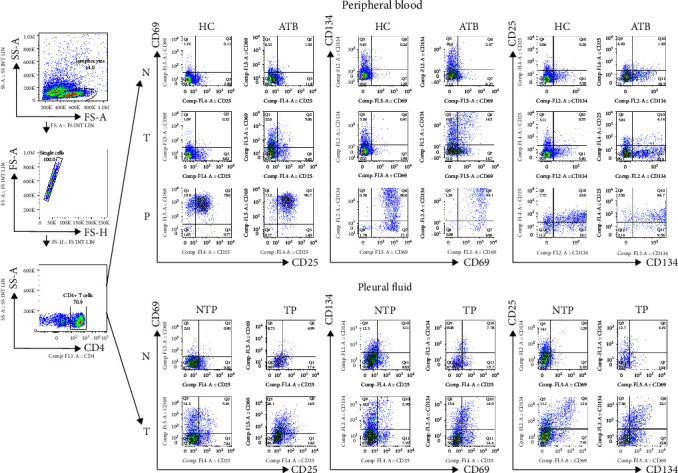
Representative flow cytometry analysis showing the gating strategy for the analyses of CD4+ T cells coexpressing CD25/CD69/CD134. Representative ATB, HC, TP, and NTP subjects were tested for CD4+ T cells coexpressing CD25/CD69/CD134 markers in negative tubes (N), test tubes (T), and positive tubes (PHA). The results of the ATB and HC group were from peripheral blood, and the results of the TP and NTP group were from pleural fluid. ATB = active tuberculosis; HC = healthy control; TP = tuberculous pleurisy; NTP = nontuberculous pleurisy.

Standard gating procedures using isotype controls were performed to identify the positive cells. The background CD25/CD69/CD134 expression values observed in the negative control tubes were subtracted from those in the test tubes.  Figure 2 shows the gating strategy used to identify CD4+ T cells expressing CD25/CD69/CD134. Test result = percentage of cells within the test tube (T) − percentage of cells within the negative control tube (N). If the percentage of cells within T was less than that within N, the test result was denoted as 0.

### 2.4. Statistical Analysis

Continuous variables were expressed as mean ± standard deviation (SD) in the case of normally distributed data, otherwise median (interquartile range) values. Differences in clinical and laboratory data between groups were compared using the Student's *t*-test or Mann–Whitney *U* test, as appropriate. Fisher's exact test or the chi-square (*χ*2) test was used for categorical data. Receiver operating characteristic (ROC) curve analysis was performed to determine the diagnostic efficacy of various indexes. The area under the curve (AUC), Jorden indicator, sensitivity, specificity, positive predictive value (PPV), negative predictive value (NPV), and accuracy, together with their 95% confidence interval (CIs), were calculated. Statistical analyses were performed using FlowJo_v10.6.2, SPSS 20.0, and GraphPad Prism 9. Statistical significance was set at *P* < 0.05.

## 3. Results

### 3.1. Characteristics of the Study Population

The demographic and clinical characteristics of the participants are presented in Table 1. In total, cohort 1 included 45 patients with ATB, 40 LTBI individuals, 50 patients with NTB, and 42 HC; and cohort 2 included 28 patients with TP and 36 patients with NTP. The mean age of the participants was 65, and more than half of them were male. In both cohorts, there were no significant differences in age or sex among the groups. In cohort 1, 62.2% of the patients with ATB were confirmed using the TB molecular method, and nearly half of the patients with ATB were Mtb culture positive. Meanwhile, the TB-IGRA positivity rates of ATB and LTBI groups were 97.8% and 100.0%, respectively. In cohort 2, 24 (85.7%) patients with TP and eight (22.2%) patients with NTP were positive for TB-IGRA.

### 3.2. Comparison of CD25, CD69, and CD134 Expression Patterns among Different Groups in Cohort 1

**Figure 3:**
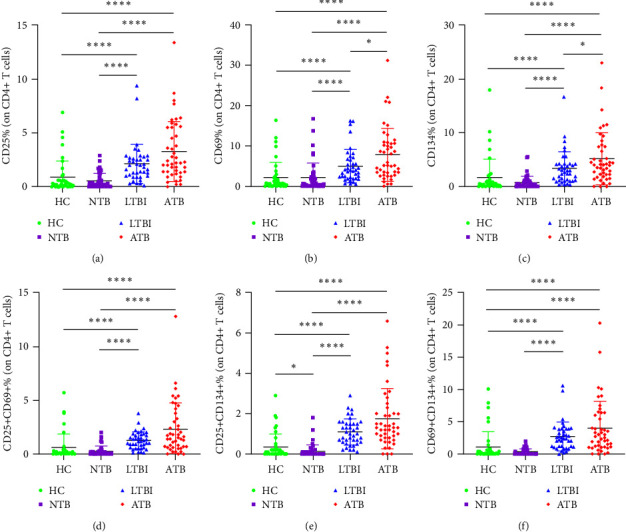
Comparison of the results of CD25, CD69, and CD134 on CD4+ T cells in PB between the groups. (a) Comparison of the results of CD25 on CD4+ T cells in PB between the groups. (b) Comparison of the results of CD69 on CD4+ T cells in PB between the groups. (c) Comparison of the results of CD134 on CD4+ T cells in PB between the groups. (d) Comparison of the results of CD25+CD69+ on CD4+ T cells in PB between the groups. (e) Comparison of the results of CD25+CD134+ on CD4+ T cells in PB between the groups. (f) Comparison of the results of CD69+CD134+ on CD4+ T cells in PB between the groups. *P* values were determined by Mann–Whitney *U* test.  ^*∗*^*P* < 0.05;  ^*∗∗∗∗*^*P* < 0.0001. PB = peripheral blood; ATB (*n* = 45) = active tuberculosis; LTBI (*n* = 40) = latent tuberculosis infection; NTB (*n* = 50) = nontuberculosis; HC (*n* = 42) = healthy control.

The expression and coexpression of CD25, CD69, and CD134 on CD4+ T cells in PB were detected after Mtb antigen stimulation. Significant differences were observed among the groups. The indexes and coexpression patterns in ATB and LTBI groups were significantly higher than those in the HC and NTB groups (*P* < 0.0001) (Figure 3). In addition, the expression of CD69 and CD134 in patients with ATB was higher than that in LTBI (*P* < 0.05) (Figure 3(b)).

### 3.3. Diagnostic Value of CD25+, CD69+, CD134+, and Double Positive CD4+ T Cells for ATB

**Figure 4:**
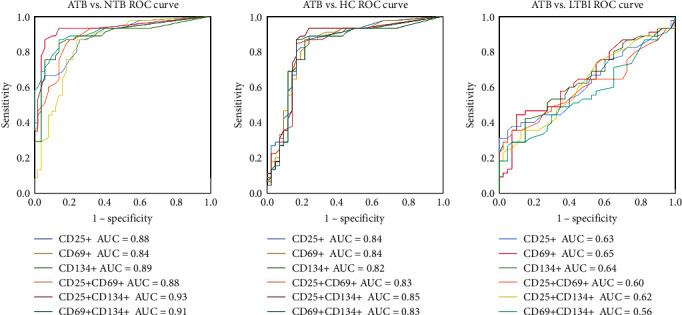
Diagnostic value of CD25+, CD69+, CD134+, and double positive CD4+ T cells in PB for ATB. PB = peripheral blood; ATB (*n* = 45) = active tuberculosis; LTBI (*n* = 40) = latent tuberculosis infection; NTB (*n* = 50) = nontuberculosis; HC (*n* = 42) = healthy control. *P* values were determined by *Z*-test. In the differential diagnosis of ATB and NTB/HC, all the *P* values of markers in PF and PB were <0.0001. In the differential diagnosis of ATB and LTBI, *P*_CD25_ = 0.05, *P*_CD69_ = 0.02, *P*_CD134_ = 0.03, *P*_CD25+CD69+_ = 0.12, *P*_CD25+CD134+_ = 0.06, *P*_CD69+CD134+_ = 0.33.

To further evaluate the diagnostic value of each PB indicator for ATB diagnosis, ROC curve analyses were performed (Figure 4). The results showed that in the differential diagnosis of ATB and NTB/HC, the AUC values of the activation indexes were all greater than 0.8. Among them, the coexpression of CD25 and CD134 on CD4+ T cells achieved an AUC of 0.93 (95% CI, 0.87–0.99) in differentiating ATB from NTB (Figure 4). When 0.5 was used as the cutoff value, the sensitivity and specificity values for distinguishing ATB from NTB were 86.7% (95% CI, 72.5%–94.5%) and 94.0% (95% CI, 82.5%–98.4%), respectively (Table 2). Nevertheless, the value of the activation indexes in distinguishing ATB from LTBI was very limited, with CD69 reaching the optimal AUC value at 0.65 (95% CI, 0.53–0.77). When the threshold was set at 8.35, CD69+CD4+ T cells showed a sensitivity of 44.4% (95% CI, 30.0%–60.0%) and a specificity of 90.0% (95% CI, 75.4%–96.8%) for ATB and LTBI discrimination (Table 2). Interestingly, compared to those of single expressions, the coexpression of the two activation indexes did not improve the differential diagnosis of ATB and LTBI but had a higher diagnostic efficiency in the differentiation of ATB and NTB (those for CD25+CD134+ and CD69+CD134+).

### 3.4. Comparison of the Diagnostic Performance of Different Activation Indexes and Routine Laboratory Methods for ATB Diagnosis

The diagnostic performance of routine laboratory methods for ATB diagnosis, including Xpert MTB/RIF, acid-fast bacilli smear, and Mtb culture, were compared with that of activation indexes. Unexpectedly, the accuracy of activation indicators in the diagnosis of ATB in this study was higher than that of conventional laboratory methods, except for TB-IGRA. Among the CD25, CD69, and CD134 expression patterns, the coexpression of CD25+CD134+ and CD69+CD134+ was the most accurate in the diagnosis of ATB (85.1%) (Table 3).

### 3.5. Comparison of TP and NTP Activation Marker Expression in PB and PF

**Figure 5:**
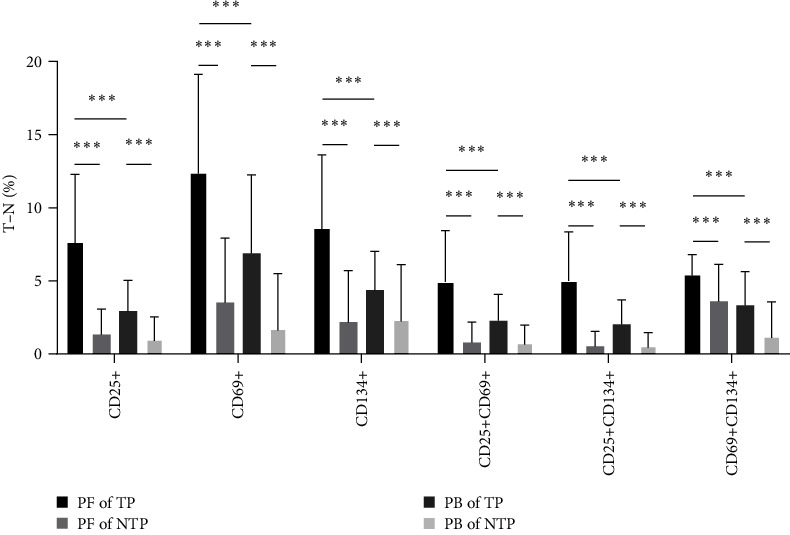
Comparison of each activation index in PB and PF between TP and NTP. Test result = percentage of cells within the test tube (T) ˗ percentage of cells within the negative control tube (N). PF = pleural fluid; PB = peripheral blood; TP (*n* = 28) = tuberculous pleurisy; NTP (*n* = 36) = nontuberculous pleurisy. Results are expressed as the mean ± SD. *P* values were determined by Mann–Whitney *U* test.  ^*∗∗∗*^*P* < 0.001.

To verify the diagnostic value of each activation indicator for the diagnosis of ATB, we further compared the expression and coexpression of different indexes in PB and PF between the TP and NTP groups (cohort 2). The expression and coexpression of activation markers in both the PB and PF of patients with TP were significantly higher than those of patients with NTP (*P* < 0.001). Compared with those of PB, the expression and coexpression of activation indexes in PF were significantly higher in patients with TP (*P* < 0.001) (Figure 5).

### 3.6. Performance of Activation Markers in PB and PF in the Differential Diagnosis of TP and NTP

**Figure 6:**
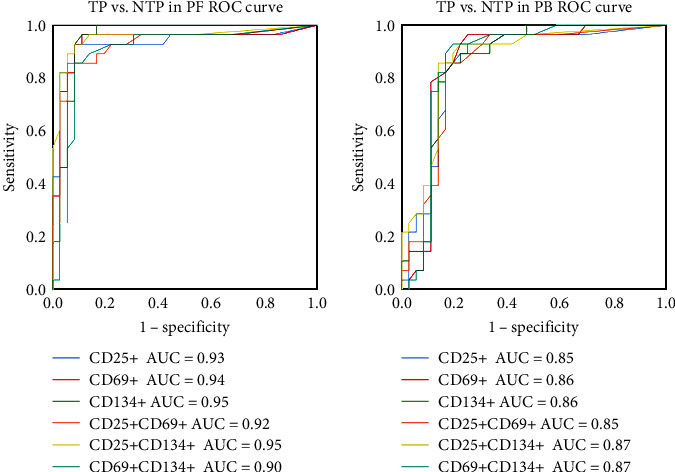
Diagnostic value of each activation index in PF and PB for TP. PF = pleural fluid; PB = peripheral blood; TP (*n* = 28) = tuberculous pleurisy; NTP (*n* = 36) = nontuberculous pleurisy. *P* values were determined by *Z*-test. All the *P* values of markers in PF and PB were <0.0001.

ROC curves were created to better evaluate the value of each activation marker and their double-positive combinations in diagnosing TP (Figure 6). Similar to the results in cohort 1, when the expression of activation indexes on CD4+ T cells in PB was used to differentiate TP and NTP, the coexpression of CD25 and CD134 showed the highest diagnostic efficacy. When 0.7 was used as the cutoff value, the sensitivity, specificity, PPV, and NPV were 85.7% (95% CI, 66.4%–95.3%), 86.1% (95% CI, 69.7%–94.8%), 82.8% (95% CI, 63.5%–93.5%), and 88.6% (95% CI, 72.3%–96.3%), respectively (Table 4). In ATB, Mtb-specific T cells are clonally expanded and recruited to the site of infection. This suggested that individual indicators and their double-positive combinations in PF would be more sensitive to the diagnosis of TP than those in PB. As expected, the AUC of each indicator and those of their coexpression combinations in PF, which were all over 0.9, were more efficient than those in PB (Table 4) in discriminating patients with TP from those with NTP (Figure 6). Among the single expression of activation markers in PF, CD134+CD4+ T cells had the highest AUC (0.95 : 95% CI, 0.90–1.00) with a sensitivity of 96.4% (95% CI, 79.8%–99.8%) and specificity of 88.9% (95% CI, 73.0%–96.4%). When using coexpression activation indexes, CD25 and CD134 coexpression achieved the highest AUC value (0.95 : 95% CI, 0.90–1.00), along with a sensitivity of 92.9% (95% CI, 75.0%–98.8%), specificity of 91.7% (95% CI, 76.4%–97.8%), PPV of 89.7% (95% CI, 71.5%–97.3%), and NPV of 94.3% (95% CI, 79.5%–99.0%).

These results suggested that the coexpression of CD25 and CD134 on CD4+ T cells in both PB and PF specimens has a good differential diagnostic value for TP and a better diagnostic efficacy in PF.

## 4. Discussion

To control TB, it would be of great significance to find a fast and accurate diagnostic method for Mtb infection. However, difficulties remain in the diagnosis of ATB and TP [35]. Currently, many immune biomarkers have been evaluated, including some activation indexes that are potentially valuable for TB diagnosis but require further clarification [24, 36]. In the present study, the expression of CD25, CD69, CD134 and their double-positive combinations on TB antigen-stimulated CD4+ T cells were detected to determine their effectiveness in ATB and TP diagnosis. The role of activation marker expression patterns in PB and PF samples in the differential diagnosis of TP and NTP was also compared. Our results showed that the expression of CD25, CD69, CD134 and their double-positive combinations on TB-reactive CD4+ T cells were valuable for the diagnosis of ATB. Moreover, the diagnostic performance of double-positive combinations was better than that of single expression profiles in the differentiation of ATB and NTB, with CD25+CD134+ showing the highest diagnostic efficiency. Except for TB-IGRA, the diagnostic accuracy of the activation indexes for ATB was higher than that of conventional laboratory methods. Interestingly, the expression of activation markers in PF was significantly higher than that in PB, especially the coexpression of CD25 and CD134 on CD4+ T cells, and the differential diagnosis of TP and NTP using PF samples was better than that using PB. Our study explored the expression profiles of different activation markers on CD4+ T cells, which provided valuable information for the diagnosis of ATB and further proved that the expression of activation markers in PF is of great significance for the diagnosis of TP.

In cohort 1 (*n* = 177), we analyzed the expression of CD25, CD69, and CD134 on CD4+ T cells among different groups to evaluate the value of different activation indexes in ATB diagnosing. Early experimental evidence, such as that from human immunodeficiency virus infection models involving adoptive transfer of CD4+ T cells [37] has proven that CD4+ T cells play a leading role in controlling Mtb infection. Zauders et al. [25] observed the upregulation of CD25 and CD134 in antigen-specific CD4+ T cells. CD25 is the alpha chain of IL-2 receptor, the upregulation of high-affinity IL-2R after stimulation of T cells is very important in controlling the proliferation, differentiation, and apoptosis of CD4+ T cells [38]. CD134 is expressed after T cell receptor participation and reaches a peak after 24–48 hr. Few CD134 molecules are expressed on unstimulated CD4+ T cells in human PB [39]. Consistent with previous studies [25, 30], we found that CD25 and CD134 expression and their coexpression in the ATB and LTBI groups were significantly higher than those in the HC group. The diagnostic efficacy of CD25 and CD134 coexpression was better than that of the individual expression of the markers for ATB and NTB identification. In addition, the percentage of CD69+CD4+ T cells in the ATB group was significantly higher than that in the LTBI group. Because CD69 is a surface antigen, first expressed after T lymphocyte activation [40], this phenomenon may be due to early immune activation in the active course of the disease. Nevertheless, ROC curve analysis showed that the value of these activation markers in distinguishing ATB from LTBI was limited.

In addition, our results showed that the diagnostic sensitivity of each activation indicator was higher than that of conventional laboratory methods. Since the percentage of CD4+ T cell activation markers is not affected by the absolute reduction of lymphocytes in patients [41], it can compensate for the false negatives of TB-IGRA due to the low absolute number of lymphocytes. This simple and safe method can be applied in clinical practice for TB diagnosis.

Reactivation is the main driver of TB pathogenesis, and pleural involvement has been reported in 4% of the cases. A survey [42] showed that the incidence of TP is higher in young patients worldwide. However, patients with both ATB and TP are relatively old in TB epidemic areas, mainly because ATB is not caused by a primary infection. The early response of the body to pleural injury due to Mtb is dominated by neutrophils, followed by a large release of macrophages and a long-term lymphocyte-driven immune response, accompanied by the formation of pleural granuloma and the release of ADA [43]. However, as the second most common form of EPTB, TP remains difficult to diagnose. The sensitivity of acid-fast bacilli smear and Mtb culture is very low [44], and other indicators, such as lymphocyte percentage and ADA level in PF, also have limited diagnostic values for TP [45]. Thus, there is an urgent need for rapid and safe methods to facilitate TP diagnosis.

Since the lymphocytes found in TB PF are mainly T helper cells, we further compared the expression of CD25, CD69, and CD134 on TB-specific CD4+ T cells in PB and PF to differentiate TP from NTP. In cohort 2 (*n* = 84), our results showed that the activation indexes of the markers in both PB and PF were valuable for distinguishing between TP and NTP. In addition, the indexes from the PF of patients with TP were higher than those from PB and showed a higher efficiency than the latter in the differential diagnosis of TP and NTP. This may be due to the enrichment of memory T cells in the TB PF, which not only increase in number but show a strong specific response after stimulation. The phenomenon describing that the proportion of T helper cells in TB PF is significantly higher than that in PB has been called “compartmentalization” [46]. Nemeth et al. [47] found that patients with ATB showed significant enrichment of Mtb-specific T cells at the infected site. Because TB antigen-specific CD4+ T cells are recruited to the infection site, PF is richer in antigen-experienced T cells than matched PB.

In ATB, Mtb-specific tissue-resident memory T cells are clonally expanded and recruited to the infected site expressing activation markers and cytokines [48]. Similar to our results, Luo et al. [49] showed that CD4+ T cells in the PF were more activated than CD4+ T cells in the PB of patients with TP. Liao et al. [50] also showed that TB-specific cells that produce IFN-*γ* were enriched in the PF of patients with TP but not in the PF of NTP individuals. Losi et al. [5] evaluated the use of ELISpot for PF diagnosis of TP; however, its specificity was inferior to that of CD25+CD134+ reported in our study. Additionally, the performance of PF T-SPOT in the differential diagnosis of TP is significantly better than that of PB T-SPOT [7], indirectly confirming the value of PF markers in TP diagnosis. However, owing to the large-scale variability in the number of lymphocytes in PF, the use of PF for T-SPOT testing is limited. In our study, sample pretreatments were carried out to achieve sufficient and relative consistency of lymphocytes before detection, facilitating standardization, and compensating for the deficiency in T-SPOT.TB detection.

Our study also has some limitations. First, the number of recruited subjects in each group was limited, and results should be further validated in larger cohorts. Second, several activation markers of CD4+ T cells have been proven to be valuable in the diagnosis of TB, but they have not been systematically evaluated in our study. Finally, we did not detect TB-reactive IFN-*γ* to accurately reflect the expression of activation indexes in TB-specific T cells. Therefore, further research is required to fill this gap.

## 5. Conclusion

In this study, the values of early (CD25 and CD69) and late (CD134) markers of lymphocyte activation in the differential diagnosis of ATB were evaluated in both PB and PF. We proposed that the expression indexes of TB-specific CD4+ T cell activation markers CD25, CD69, and CD134 could be promising new tools for the diagnosis of ATB, with the coexpression of CD25 and CD134 optimal for differential diagnosis. In addition, T cell activation indexes in PF are more valuable in diagnosing TP than those in PB. In summary, the activation indexes in this study have good diagnostic efficacy for ATB diagnosis, especially for TP, and are expected to be applied in the clinic.

## Figures and Tables

**Table 1:** Clinical and laboratory characteristics of study participants.

Variables	Cohort 1				Cohort 2	
	ATB (*n* = 45)	LTBI (*n* = 40)	NTB^b^ (*n* = 50)	HC (*n* = 42)	TP (*n* = 28)	NTP (*n* = 36)
Age, years	70.0 (51.0–86.0)	70.5 (51.0–86.8)	70.0 (50.3–89.0)	59.5 (52.8–70.0)	62.5 (53.5–68.0)	63.5 ± 11.4
Gender, male (*n*, %)	25 (55.6%)	24 (60.0%)	27 (54.0%)	28 (66.7%)	19 (67.9%)	25 (69.4%)
Radiological findings^a^ (*n*, %)	8 (17.8%)	4 (10.0%)	0	0		
Acid-fast bacilli smear positive (*n*, %)	20 (44.4%)	0	0	0	2 (7.1%)	0
Xpert MTB/RIF positive (*n*, %)	28(62.2%)	0	0	0	7 (25.0%)	0
Mtb culture positive (*n*, %)	22 (48.9%)	0	0	0	2 (7.1%)	0
TB-IGRA positive (*n*, %)	44 (97.8%)	40(100.0%)	0	0	24 (85.7%)	8 (22.2%)
ADA (U/L)	NA	NA	NA	NA	44.6 (34.2–55.4)	11.9 (7.6–21.0)

*Note*: ATB = active tuberculosis; LTBI = latent tuberculosis infection; NTB = nontuberculosis; HC = healthy control; TP = tuberculous pleurisy; NTP = nontuberculous pleurisy; Mtb = *Mycobacterium tuberculosis*; NA = not applicable. ^a^Lung shadow or pathological changes. ^b^Disease type of NTB: chronic disease, 19 (38.0%); infectious diseases, 14 (28.0%); autoimmune diseases,12 (24.0%); tumors, 5 (10.0%).

**Table 2:** The diagnostic value of each activation index in PB for ATB.

Variables	AUC (95% CI)	Youden indicator	Cutoff value	Sensitivity (95% CI)	Specificity (95% CI)	PPV (95% CI)	NPV (95% CI)	Accuracy (%)
ATB vs. NTB								
CD25+	0.88 (0.82–0.95)	0.6	0.9	86.7% (72.5%–94.5%)	74.0% (59.4%–84.9%)	75.0% (60.8%–85.5%)	86.0% (71.4%–94.2%)	80.0
CD69+	0.84 (0.76–0.92)	0.6	2.4	86.7% (72.5%–94.5%)	76.0% (61.5%–86.5%)	76.5% (62.2%–86.8%)	86.4% (72%–94.3%)	81.1
CD134+	0.89 (0.81–0.96)	0.7	1.4	84.4% (69.9%–93.0%)	86.0% (72.6%–93.7%)	84.4% (69.9%–93%)	86.0% (72.6%–93.7%)	85.3
CD25+CD69+	0.88 (0.81–0.95)	0.7	0.4	86.7% (72.5%–94.5%)	80.0% (65.9%–89.5%)	79.6% (65.2%–89.3%)	87.0% (73.1%–94.6%)	83.2
CD25+CD134+	0.93 (0.87–0.99)	0.8	0.6	86.7% (72.5%–94.5%)	94.0% (82.5%–98.4%)	92.9% (79.5%–98.1%)	88.7% (76.3%–95.3%)	90.5
CD69+CD134+	0.91 (0.85–0.97)	0.7	0.9	86.7% (72.5%–94.5%)	86.0% (72.6%–93.7%)	84.8% (70.5%–93.2%)	87.8% (74.5%–94.9%)	86.3
ATB vs. HC								
CD25+	0.84 (0.76–0.93)	0.7	0.8	86.7% (72.5%–94.5%)	78.6% (62.8%–89.2%)	81.3% (66.9%–90.6%)	84.6% (68.8%–93.6%)	82.8
CD69+	0.84 (0.75–0.93)	0.7	2.3	86.7% (72.5%–94.5%)	78.6% (62.8%–89.2%)	81.3% (66.9%–90.6%)	84.6% (68.8%–93.6%)	82.8
CD134+	0.82 (0.72–0.92)	0.7	1.1	88.9% (75.2%–95.8%)	76.2% (60.2%–87.4%)	80.0% (65.9%–89.5%)	86.5% (70.4%–94.9%)	82.8
CD25+CD69+	0.83 (0.74–0.92)	0.7	0.5	84.4% (69.9%–93%)	83.3% (68.0%–92.5%)	84.4% (69.9%–93%)	83.3% (68.0%–92.5%)	83.9
CD25+CD134+	0.85 (0.75–0.94)	0.7	0.6	86.7% (72.5%–94.5%)	83.3% (68.0%–92.5%)	84.8% (70.5%–93.2%)	85.4% (70.1%–93.9%)	85.1
CD69+CD134+	0.83 (0.74–0.93)	0.7	0.9	86.7% (72.5%–94.5%)	83.3% (68.0%–92.5%)	84.8% (70.5%–93.2%)	85.4% (70.1%–93.9%)	85.1
ATB vs. LTBI								
CD25+	0.63 (0.51–0.74)	0.3	4.4	31.1% (18.6%–46.8%)	100.0% (89.1%–100.0%)	100.0% (73.6%–100.0%)	56.3% (44.1%–67.9%)	56.8
CD69+	0.65 (0.53–0.77)	0.3	8.4	44.4% (30.0%–60.0%)	90.0% (75.4%–96.8%)	83.3% (61.8%–94.5%)	59.0% (45.7%–71.2%)	59.0
CD134+	0.64 (0.52–0.76)	0.3	4.4	42.2% (28.0%–57.8%)	85.0% (69.5%–93.8%)	76.0% (54.5%–89.8%)	56.7% (43.3%–69.2%)	55.8
CD25+CD69+	0.60 (0.48–0.72)	0.3	2.3	35.6% (22.3%–51.3%)	92.5% (79.5%–98.0%)	84.2% (59.5%–95.8%)	56.1% (43.4%–68.1%)	55.8
CD25+CD134+	0.62 (0.50–0.74)	0.2	1.9	35.6% (22.3%–51.3%)	87.5% (72.4%–95.3%)	76.2% (52.5%–90.9%)	54.7% (41.8%–70.0%)	53.7
CD69+CD134+	0.56 (0.44–0.69)	0.2	5.7	26.7% (15.1%–42.2%)	95.0% (81.8%–99.1%)	85.7% (56.2%–97.5%)	53.5% (41.4%–65.3%)	52.6

*Note*: PPV = positive predictive value; NPV = negative predictive value; PB = peripheral blood; ATB (*n* = 45) = active tuberculosis; LTBI (*n* = 40) = latent tuberculosis infection; NTB (*n* = 50) = nontuberculosis; HC (*n* = 42) = healthy control.

**Table 3:** The diagnostic efficacy of activation indexes and routine laboratory methods for ATB diagnosis.

Variables	Sensitivity (95% CI)	Specificity (95% CI)	PPV (95% CI)	NPV (95% CI)	Accuracy (%)
Xpert MTB/RIF	62.2% (46.5%–75.8%)	100.0% (89.6%–100.0%)	100.0% (85%–100.0%)	71.2% (57.7%–82.0%)	80.5
Acid-fast bacilli smear	44.4% (30.0%–59.9%)	100.0% (89.6%–100.0%)	100.0% (98%–100.0%)	62.7% (50.0%–73.9%)	71.3
Mtb culture	48.9% (33.9%–64.0%)	100.0% (89.6%–100.0%)	100.0% (81.5%–100.0%)	64.6% (51.7%–75.8%)	73.6
TB-IGRA	95.6% (83.6%–99.2%)	90.5% (76.5%–96.9%)	91.5% (78.7%–97.2%)	95.0% (81.8%–99.1%)	93.1
CD25+	86.7% (72.5%–94.5%)	78.6% (62.8%–89.2%)	81.3% (66.9%–90.6%)	84.6% (68.8%–93.6%)	82.8
CD69+	86.7% (72.5%–94.5%)	78.6% (62.8%–89.2%)	81.3% (66.9%–90.6%)	84.6% (68.8%–93.6%)	82.8
CD134+	88.9% (75.2%–95.8%)	76.2% (60.2%–87.4%)	80.0% (65.9%–89.5%)	86.5% (70.4%–94.9%)	82.8
CD25+CD69+	84.4% (69.9%–93.0%)	83.3% (68.0%–92.5%)	84.4% (69.9%–93%)	83.3% (68.0%–92.5%)	83.9
CD25+CD134+	86.7% (72.5%–94.5%)	83.3% (68.0%–92.5%)	84.8% (70.5%–93.2%)	85.4% (70.1%–93.9%)	85.1
CD69+CD134+	86.7% (72.5%–94.5%)	83.3% (68.0%–92.5%)	84.8% (70.5%–93.2%)	85.4% (70.1%–93.9%)	85.1

**Table 4:** Comparison of diagnostic efficacy of PF and PB in the diagnosis of TP.

Variables	AUC (95% CI)	Youden Indicator	Cutoff value	Sensitivity (95% CI)	Specificity (95% CI)	PPV (95% CI)	NPV (95% CI)	Accuracy (%)
PF								
CD25+	0.93 (0.86–1.00)	0.9	1.9	92.9% (75.1%–98.8)	91.7% (76.4%–97.8%)	89.7% (71.5%–97.3%)	94.3% (79.5%–99.0%)	92.2
CD69+	0.94 (0.87–1.00)	0.85	3.4	96.4% (79.7%–99.8%)	88.9% (73.0%–96.4%)	87.1% (69.2%–95.8%)	97.0% (82.5%–99.8%)	92.2
CD134+	0.95 (0.90–1.00)	0.9	2.1	96.4% (79.8%–99.8%)	88.9% (73.0%–96.4%)	87.1% (69.2%–95.8%)	97.0% (82.5%–99.8%)	92.2
CD25+CD69+	0.92 (0.85–1.00)	0.8	1.7	85.7% (66.4%–95.3%)	91.7% (76.4%–97.8%)	88.9% (69.7%–97.1%)	89.2% (73.6%–96.5%)	89.1
CD25+CD134+	0.95 (0.90–1.00)	0.9	1.3	92.9% (75.0%–98.8%)	91.7% (76.4%–97.8%)	89.7% (71.5%–97.3%)	94.3% (79.5%–99.0%)	92.2
CD69+CD134+	0.90 (0.81–0.99)	0.8	2.2	85.7% (66.4%–95.3%)	91.7% (76.4%–97.8%)	88.9% (69.7%–97.1%)	89.2% (73.6%–96.5%)	89.1
PB								
CD25+	0.85 (0.75–0.95)	0.7	1.1	85.7% (66.4%–95.3%)	83.3% (66.5%–93.0%)	80.0% (60.9%–91.6%)	88.2% (71.6%–96.2%)	84.4
CD69+	0.86 (0.76–0.96)	0.7	2.3	89.3% (70.6%–97.2%)	77.8% (60.4%–89.3%)	75.8% (57.4%–88.3%)	90.3% (73.1%–97.5%)	82.8
CD134+	0.86 (0.76–0.96)	0.7	0.9	96.4% (79.8%–99.8%)	75.0% (57.5%–87.3%)	75.0% (57.5%–87.3%)	96.4% (79.8%–99.8%)	84.4
CD25+CD69+	0.85 (0.75–0.95)	0.7	0.8	85.7% (66.4%–95.3%)	83.3% (66.5%–93.0%)	80.0% (60.9%–91.6%)	88.2% (71.6%–96.2%)	84.4
CD25+CD134+	0.87 (0.78–0.96)	0.7	0.7	85.7% (66.4%–95.3%)	86.1% (69.7%–94.8%)	82.8% (63.5%–93.5%)	88.6% (72.3%–96.3%)	85.9
CD69+CD134+	0.87 (0.77–0.97)	0.7	0.7	92.9% (75.0%–98.8%)	80.6% (63.4%–91.2%)	78.8% (60.6%–90.4%)	93.6% (77.2%–98.9%)	85.9

*Note*: TP (*n* = 28) = tuberculous pleurisy; PF = pleural fluid; PB = peripheral blood.

## Data Availability

The data used to support the findings of this study were included in the article.
